# Promoting immune defensive responses of epithelial cells in airway disease

**DOI:** 10.3389/falgy.2025.1697194

**Published:** 2025-10-30

**Authors:** Michael J. Parnham, Virginia Norris, Jennifer A. Kricker

**Affiliations:** EpiEndo Pharmaceuticals, Reykjavik, Iceland

**Keywords:** collectins, SP-D, SP-A, epithelium, alarmins, host defense, respiratory

## Abstract

The airway epithelium serves as both a physical barrier and as an active contributor in maintaining immune defense. Upon exposure to external insults such as injury and infection, the epithelium releases alarmins including interleukin-25 (IL-25), IL-33, and thymic stromal lymphopoietin (TSLP), which assist in initiating and amplifying the immune response. Complementing these are the collectins, particularly surfactant protein-D (SP-D), which also participate in the innate immune response. SP-D along with its closely related collectin, SP-A, bind pathogens, apoptotic cells, and allergens, promoting phagocytosis while modulating inflammation and preventing excessive Th2-driven responses. This review discusses the role of the airway epithelium in host defense mechanisms, particularly in chronic obstructive pulmonary disease (COPD) and asthma, and explores the therapeutic implications of epithelial-driven immune responses in respiratory inflammation.

## 1 Introduction

The airway epithelium plays a crucial role in maintaining lung homeostasis, serving as both a physical barrier and an active participant in immune defense. In response to external stimuli, it releases epithelial-derived alarmins that initiate a cascade of immune responses, a topic that has received intensive consideration in recent years. This present review discusses the role of the airway epithelium in host defense mechanisms, particularly in COPD and asthma, and explores the therapeutic implications of epithelial-driven immune responses in respiratory inflammation. In particular, we address the roles of collectins and their complementary roles to those of alarmins. Although many factors contribute to both homeostasis and the development and chronicity of airway diseases, including members of the interferon (IFN) family, we focus on the roles of collectins and how they interplay with those key alarmins.

## 2 When is a collectin an alarmin?

### 2.1 Alarmins

Functioning as danger signals, alarmins or damage-associated molecular patterns (DAMPs), are a diverse group of endogenous molecules known to signal the immune system upon threats and damage to cells and tissues. Generally, alarmins are defined by their release upon cellular stress or damage; their capacity to initiate or amplify the innate and adaptive immune responses; and to participate in repair and homeostasis (1). As they are grouped by function rather than physical characteristics, they can vary greatly in their molecular weights and structures. This is reflected in the types of alarmins, ranging from proteins to metabolites to nucleic acids, which meet the criteria to varying degrees (2).

In the lung, key epithelial-derived alarmins include interleukin-25 (IL-25), IL-33, TSLP, and high mobility group box-1 (HMGB-1) (Table 1). These molecules are central to type-2 immune responses, although they may act on a broader spectrum of cells. Epithelial cells are the primary source of IL-25, TSLP and IL-33 and often released upon injury, but these alarmins can also be produced by immune cells, endothelial cells and fibroblasts under certain conditions (3).

**Table 1:** Source and roles of collectins and alarmins during airway inflammation.

Feature	Collectins (SP-A, SP-D)	Alarmins (IL-25, IL-33, TSLP)
Source	Alveolar type II, club cells; bronchial and extrapulmonary epithelia	Airway and nasal epithelium; endothelial cells, fibroblasts, immune cells
Release	Constitutively secreted into airway surface fluid;	Injury, oxidative stress, infection, necrosis, viral or allergen stimulation
	Injury, infection, oxidative stress	
Act Upon	CD91 → phagocytosis	IL-25 → activates ILC2 → IL-5, IL-13 leading to eosinophilia, mucus
	CD14 and TLR to modulate action of LPS	
		IL-33 binds ST2 receptor on mast cells, DCs, Th2 resulting in a cytokine storm (IL-4, IL-5, IL-13)
	Binds to PAMPs	
	Bind to and agglutinate pathogens and modify IgE/allergen interaction	TSLP primes DCs, mast cells, ILC2 leading to Th2 polarization
	Inhibit allergen induced eosinophil, basophil and mast cell degranulation	
	Modify DC activation and inhibit T cell proliferation	
Immune Defense	SP-D binds SIRP*α* & suppresses NF*κ*B → anti-inflammatory	Amplify innate & adaptive immune responses (ILC2 survival, Th2 skewing)
	SP-A promotes M2 macrophages → phagocytosis of pathogens/apoptotic cells	Crosstalk with GM-CSF (antiviral)
	Promote pathogen and allergen clearance	
	Inhibition of allergen-induced responses	
Modifications	Gene polymorphisms	Oxidized IL-33 → binds to RAGE/EGFR
	Monomeric and multimeric forms	
Fate	Cleaved by MMP-9, NE, ROS → loss of function	Degradation by caspases and proteolytic enzymes including NE

IL-25, also known as IL-17E, is a member of the IL-17 cytokine family that is widely distributed and produced by CD4+ and CD8+ Th2 cells and tuft cells of the respiratory and intestinal tracts, as well as a variety of immune, epithelial and endothelial cells, and fibroblasts (4). IL-25 production and secretion occur in response to pathogens, allergens, and cellular stress, resulting in it being called a “barrier surface” cytokine due to its response to external damage to epithelial cells. Actions of IL-25 occur via its receptor, a heterodimeric cell surface receptor comprising IL17RA and IL17RB. Similar to other alarmins, downstream signaling involves NF-*κ*B and mitogen-activated protein kinase (MAPK), along with JAK/STAT, leading to diverse responses related to allergy, autoimmune diseases, and cancer tumorigenesis (4–6).

IL-33, a member of the IL-1 cytokine family, is produced as a biologically active full-length nuclear protein, primarily by epithelial and endothelial cells (7–9). Under homeostatic conditions, IL-33 remains in the nucleus and may play a role in regulatory functions. Upon cell or tissue damage, however, IL-33 is released extracellularly and undergoes enzymatic cleavage enabling it to interact with the heterodimeric ST-2 receptors (also known as IL1RL1), mainly found on immune cells, including dendritic cells (DCs), mast cells, eosinophils, and macrophages. Binding leads to formation of ternary complexes, triggering Myd88 pathway signaling and NF-κB and MAPK activation, ultimately resulting in Th2 cytokine production (10, 11). As well as contributing to IL-13, IL-4 and IL-5 production in allergic disease, IL-33 appears to be more pleiotropic than first thought, as recent evidence indicates its involvement in Th1 and Th17 responses, dependent upon the microenvironment (12, 13).

TSLP, a member of the IL-2 family, is rapidly upregulated and released by alveolar epithelial cells (AECs) following injury or by cytokine stimulation (e.g., IL-1β, TNF-α). The TSLP gene encodes two transcripts, a long (lfTSLP) and a short form of TSLP (sfTSLP), both expressed by human bronchial epithelial cells, but which have opposing immune functions (14, 15). While sfTSLP is constitutively expressed, lfTSLP is the isoform of interest, as it is upregulated upon injury or inflammatory stimuli. As a secretory protein, lfTSLP exerts its effects by binding to the heterodimeric receptor composed of TSLPR and IL-7a, primarily located on DCs, type 2 innate lymphoid cells (ILC2), and mast cells. Upon high affinity binding to TSLPR, it is proposed that TSLP undergoes an allosteric conformation change, allowing for recruitment of IL-7a (16).

### 2.2 Collectins

Collectins are a family of carbohydrate-binding proteins that are components of lung surfactant lining the airways and play a role in immune surveillance by binding to pathogen-associated molecular patterns (PAMPs) on pathogens and apoptotic cells. A total of nine collectins with similar structural and biological functions have been described, including organ-specific molecules, but the surfactant proteins (SPs) are most important for the lung (17).

Major airway immune protective collectins are the lectins, surfactant proteins A (SP-A) and D (SP-D), which also bind to nucleic acids, phospholipids and non-glycosylated proteins, creating a broad specificity for PAMPs. They are synthesized as monomers and assemble as trimeric subunits which combine into multimeric molecules, all of which can be detected after release. A common polymorphism in the SP-D gene results in an alteration of the distribution into multimeric or monomeric forms (18). Two other collectins, SP-B and SP-C are highly hydrophobic, interacting with surfactant phospholipids to increase surface tension and enhance the protective nature of the epithelial barrier (19). In the lung, the collectins are synthesized predominantly by alveolar type II (AT-II) cells; the phospholipid, mainly phosphatidylcholine (PC) with phosphatidyl-inositol (PI) and –glycerol (PG), and SP-B and SP-C components of lung surfactant being synthesized separately and packaged into the lamellar bodies (20, 21). SP-A and SP-D are secreted independently of lamellar bodies and contribute to immune modulation by agglutinating and opsonizing pathogens, facilitating phagocytosis, particularly by alveolar macrophages and regulating inflammation (21–23). A similar role is played by the galectins, particularly galectins 3 and 9, lectins that are distributed throughout the body and are generated on pathogen exposure from membrane and cytoplasmic sites of various cells, including epithelial cells (24). In fact, in the lung, epithelial collectins, primarily SP-A and SP-D, are produced by airway epithelial cells, alveolar type II cells, and club cells, they are also both generated from extra-pulmonary sites and contribute to epithelial first line defense mechanisms (17, 25).

Both SP-A and SP-D bind to a wide variety of protein acceptors and protein receptors (26, 27). The actions of SP-A and SP-D in promoting macrophage phagocytosis are mainly related to their binding, by dint of their similar collagenous regions, to the complement C1q receptor calreticulin which, when bound to cell surface CD91, mediates phagocytosis of micro-organisms (17). In contrast to SP-A, SP-D also binds to late apoptotic cells, facilitating their clearance by phagocytes (27). As a result, the collectins help clear both pathogens and cell debris from the lungs. Both SP-A and SP-D also bind to CD14, the receptor for bacterial lipopolysaccharide (LPS), modulating the action of LPS (28). SP-D targets core terminal saccharides, while SP-A preferentially targets lipid A in the LPS molecule (17). Trimeric subunits of SP-D exhibit greater binding to LPS and lipoproteins, while multimeric molecules are more effective at enhancing phagocytic responses of leucocytes (18, 29).

SP-D further regulates immune and inflammatory responses by binding to signal regulatory protein *α* (SIRPα) on myeloid cells which inhibits the generation of pro-inflammatory cytokines. This involves recruitment of the protein tyrosine phosphatases SHP-1 and SHP-2 which dephosphorylate key signaling pathways in the cells, promoting an inflammation resolving environment (27). Additionally, SP-D plays a modulatory role in allergic reactions, binding to allergens, and preventing their binding to IgE, as well as facilitating conversion of Th2 to Th1 responses. It also inhibits histamine release by basophils and eosinophils and promotes apoptosis in activated eosinophils, while SP-A inhibits dendritic cell DC maturation (27). Shamim et al. (27) proposed that SP-D plays a crucial immune surveillance role in the lung, bridging between innate and adaptive immunity.

Collectins interact with alarmins to fine-tune immune responses. Such examples include the modulation of IL-33 by SP-D in limiting excessive inflammation. The converse also is true, alarmins such as TSLP can influence collectin production, enhancing epithelial defense mechanisms. Understanding these interactions provides insights into the balance between protective immunity and chronic inflammation in airway diseases.

These actions are discussed in further detail in later sections (Table 1).

## 3 Immunobiology of epithelial collectins and alarmins

### 3.1 Priming the immune response

**Figure 1:**
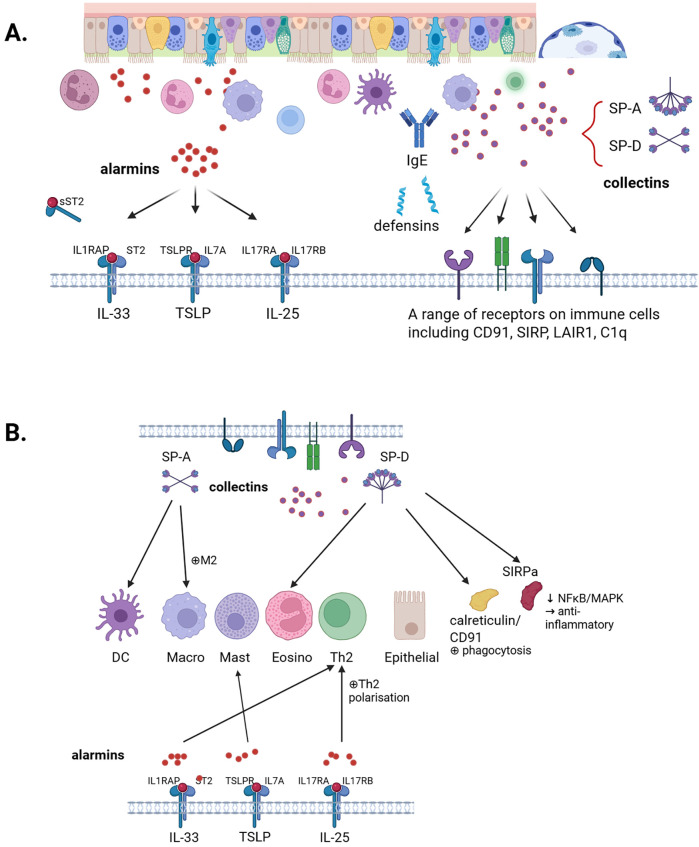
Overview of collectins and alarmins and their interactions. **(A)** This review discusses the role of the airway epithelium in host defence mechanisms, with particular emphasis on collectins, SP-A and SP-D, and alarmins IL-33, TSLP, and IL-25. **(B)** Interplay between collectins and alarmins. Created in BioRender. Kricker, J. (2025) https://BioRender.com/hklcxao.

Beyond serving as a barrier, clearly the airway epithelium actively modulates immune responses, providing a first line of defense against external pathogens and environmental insults. Initial exposure to injurious particles and infectious pathogens must first overcome ciliary clearance and mucous membrane protection, supported by production of mucins, including MUC5AC and MUC5B generated by epithelial goblet cells, and IgA produced by sub-epithelial plasma cells, which are expressed on the luminal surface to reduce pathogen adherence (30). Subsequently, a proposed five-phase overlapping lung immune defense response to external pathogens is activated (31). This involves, first, surfactant generation of collectins and phospholipids which help recognize viral, bacterial and fungal pathogens and activate initial adaptive immune responses with minimal epithelial damage. Several surfactant phospholipids, such as dipalmitoyl-PC, dioleoyl-PI and palmitoyl-oleoyl-PG bind directly to various RNA viruses and pathogenic bacteria, inhibiting their binding to pattern recognition receptors (PRR), including Toll-like receptors (TLRs) (31, 32) (Figure 1).

Subsequent phases involve type III, then type I, followed by type II IFN release, and ultimately by antibody generation, each with increasing risk of epithelial injury (31). Type I and III IFNs are generated predominantly by epithelial cells. Released on infection by respiratory viruses, these IFNs act on their respective receptors on neighboring epithelial cells to activate expression of IFN-stimulated genes (ISGs) through JAK/STAT signaling pathways, establishing an antiviral state in the cells (33). Concomitantly, under activation by infectious agents of PRRs, such as TLRs, NOD-like receptors (NLRs), RIG-I-like receptors (RLRs), C-type lectin receptors (CLRs), and formyl-peptide receptors (FPRs), alarmins and other mediators, including antimicrobial and chemotactic cathelicidin and defensin proteins are generated to further mobilize cells of the innate immune response and facilitate phagocytosis. The ultimate phagocytosis and killing of pathogens by leucocytes are supported by generation of reactive oxygen species (ROS) by the airway epithelial cells (34). During acute infections, these mechanisms are coordinated to reduce epithelial injury.

### 3.2 Collectins

While the generation of SP-A and SP-D is low in the healthy lung, acute lung injury (e.g., LPS, viruses, allergens) markedly increases the generation of these collectins (23, 27, 35). By binding to pathogens and—depending on the organism—facilitating their phagocytosis and clearance by resident alveolar macrophages and neutrophils which infiltrate in response to the infectious insult, collectins provide an important initial defense against infection (23, 27). SP-A and SP-D have also been found to directly kill some Gram-negative organisms by increasing their membrane permeability (36). Binding of SP-A and SP-D to CD14 downregulates the inflammatory actions of bacterial LPS and SP-D is reported to stimulate chemotaxis of alveolar macrophages which phagocytose the pathogens (35). Both collectins also bind to and facilitate clearance of viruses, including influenza A, respiratory syncytial virus (RSV), coronaviruses and human immunodeficiency virus, promoting immune defense reactions, each collectin in complementary ways (23). SP-D has in fact been shown to bind to alpha 2 macroglobulin (A2M), enhancing the bacterial agglutination capacity of SP-D (37). Although SP-A and SP-D bind to influenza virus and RSV in slightly different ways, they both clear the viruses and modulate proinflammatory cytokine release from the lung. Promotion of phagocytosis of collectin-bound pathogens involves binding of the collagen chains of SP-A and SP-D to CD53 (CR-1), CD93 (C1qR) and CD91/reticulin complex on neutrophils, monocytes and macrophages. Binding of SP-A and SP-D to CD91/reticulin complex and TLR-4 also stimulates pro-inflammatory cytokine release to support pathogen removal (27, 38). Effects on allergic responses are discussed below.

SP-A and SP-D also modulate monocyte and macrophage function. Once binding of pathogen and the associated collectin, for instance via CD91/reticulin, has occurred and the receptor is occupied, the collectin can then bind to SIRP*α* and the protein tyrosine phosphatases SHP-1 and SHP-2 are recruited, which dephosphorylate key signaling molecules in the NF-κB and MAPK pathways leading to inhibition of proinflammatory responses and facilitating resolution of inflammation (23, 39). SP-A also binds to IFN-γ (but not type I IFN), inhibiting its classical macrophage activating actions while binding to the CD206 mannose receptor to promote an M2 resolving macrophage phenotype (23, 40). Binding of collectins to leukocyte associated Ig-like receptor-1 (LAIR1) on monocytes inhibits the release of tissue injurious ROS (38, 41). Consequently, in addition to promotion of pathogen clearance, SP-A and SP-D also facilitate subsequent inflammation resolution, thereby reducing bystander injury during infections.

The effects of SP-A and SP-D on DCs differ. In murine bone marrow-derived DCs, SP-D bound to *Escherichia coli* enhanced the association of the bacteria to the DCs (42). In the same cells, SP-A inhibited expression of major histocompatibility complex class II and CD86 receptors also inhibiting the allostimulation by DCs of T cells and enhancing dextran endocytosis (43). This indicates that SP-A and SP-D play slightly different roles in antigen presentation.

Collectins also exert multiple actions on immune cell responses to allergens (23). Thus, SP-A and SP-D bind to allergenic extracts derived from pollen, dust mite, and *Aspergillus fumigatus*, enhancing their phagocytic clearance and alveolar macrophage generation of IFN-γ and IL-12 (44, 45). As with *E.coli* bacteria, described above, SP-D, not SP-A, also enhanced ovalbumin antigen presentation by murine DCs to ovalbumin-specific MHC II T cell hybridomas (42). Once sensitization to allergen has already occurred, however, responses to SP-A and SP-D in macrophages, now likely to have changed their phenotype, change with the activation state of the cells. Both SP-A and SP-D inhibited markers of macrophage activation, in cells from sensitized mice, possibly by acting on allergen-enhanced TLR4 expression (44, 46). For instance, SP-D suppressed allergen-stimulated macrophage NO production (47). Thus, while enhancing allergen phagocytosis and presentation to T cells, the collectins subsequently limit potential bystander inflammation by promoting a resolving macrophage phenotype.

SP-A and SP-D also inhibit specific IgE—allergen interactions, and block the allergen induced degranulation and histamine release from basophils, eosinophils and mast cells (23, 48–50). SP-A also inhibits eosinophilic IL-8 production (51). Moreover, SP-A and SP-D both modify T cell responses, inhibiting antigen and mitogen-induced proliferation, T cell activation and IFN-γ production, as well as decreasing allergen induced IgE production by B cells (23). SP-D preferentially inhibits Th2 cell responses (44). As a result, rhSP-D has been found to inhibit allergic inflammation in house dust mite (HDM)-sensitized mice (47) and both proteins reduce the proliferation of peripheral blood mononuclear cells from dust mite-sensitive children (52). Thus, the two collectins facilitate allergen clearance, but modulate the resulting inflammatory responses and promotion of type II adaptive immunity.

By recognizing epithelial PAMPs, collectins themselves may be seen as epithelial surface and soluble PRRs, since they modulate a variety of innate immune cell responses. However, unlike most membrane bound PRRs, such as TLRs, which trigger epithelial cell signaling, collectins on the one hand, facilitate phagocytosis and complement activation by pathogens, while on the other, modulating immune cell signaling. In view of their varied and complementary actions on both innate and adaptive immune responses, SP-A and SP-D have been referred to as dual functioning molecules, acting to facilitate antiviral defense, as well as exerting immunomodulatory actions (23).

### 3.3 Alarmins

As highlighted in section 2.1, alarmins have dual roles. The value of IL-33 generation in response to respiratory virus infection, however, is unclear. This is because of its potent promotion of type 2 cytokines, which polarize immune cells to a type 2 asthma-like phenotype and suppress epithelial IFN generation, which is required to promote defense against viruses (53). This caveat would also apply to IL-25, which also suppresses epithelial antiviral innate immunity (54). At least for IL-33, its interaction with GM-CSF may maintain some degree of antiviral defense, as discussed later. Other alarmins, however, can trigger type 1 immune reactions, like HMGB1 and methyl-CpG binding domain protein 2 (MBD2), which activate DCs to produce IL-12, and pro-inflammatory cytokines like IL-1β, IL-6, and IL-2 and chemokines such as CXCL8, CCL2, and GM-CSF, which maintain innate immune defense against pathogens.

## 4 Host defense in COPD and asthma

### 4.1 Epithelial barrier dysfunction

Epithelial integrity is compromised following sub-acute or chronic exposure to noxious agents such as cigarette smoke, ozone and particulate pollution, as in COPD, also aggravating and promoting allergic airway conditions, particularly in children (55). The breakdown of the barrier and its subsequent dysfunction in COPD and asthma, and even chronic rhinosinusitis with nasal polyps, can be described in terms of successive events, promoted by ongoing exposure to toxic stimuli (56, 57). Raby et al. (56), propose a vicious cycle of epithelial cell injury and immune activation, driven particularly by airway pollutants which enhance epithelial oxidative injury, loss of antioxidant defense mechanisms, DNA breaks and accelerated senescence and abnormal repair mechanisms that facilitate dysfunctional airway epithelium in asthma.

Loss of barrier integrity reflects dysfunctional tight and adherens junctions, which are essential for maintaining airway barrier homeostasis by regulating paracellular transport of ions and molecules, and cell adhesion and signaling (56, 58). Altered tight junction protein expression of claudins and occludin, as a result of pollutants such as cigarette smoke or pathogens, lead to leakage and disrupted cell signaling (59). These changes that occur in either the protein itself or at the gene level ultimately lead to inflammation and the pathogenesis of various respiratory diseases. Not only do changes in apical junctions drive subsequent disrupted homeostasis, but the alarmin HMGB1 can downregulate tight junction proteins and impair cell permeability (60), ultimately contributing to lung epithelial dysfunction.

In both COPD and asthma, there is loss of epithelial barrier function, with reduced ciliary cell differentiation and increased detachment, increased epithelial growth factor receptor (EGFR) and tendency towards fibrosis and profound loss of junction proteins, resulting in increased permeability (56). Both IL-4 and IL-13, important mediators of allergic inflammation inhibit surface expression of ZO-1, occludin, E-cadherin, and β-catenin in bronchial epithelial cells (61, 62). In allergic airway inflammation, as in asthma, IL-13 can reduce the expression of claudin-18, further impacting epithelial barrier function, as reflected in decreased claudin-18 in airway brushings from asthmatic patients (63). Some viruses, including RV and RSV are also able to promote tight junction injury, partially via ROS generation, an action which is more pronounced in bronchial epithelial cells from children with asthma (64).

Mucociliary clearance is disturbed in both COPD and asthma, leading to damage of ciliary cells, and thereby reducing the removal of pathogens. While differing slightly in the two conditions, epithelial basal cell differentiation is attenuated and goblet cell hyperplasia develops in COPD. However, released ROS and oxidation processes damage tight junction proteins and disrupt epithelial integrity, thus increasing permeability of the barrier in both diseases. Epithelial cytokines and alarmins are released, recruiting inflammatory cells and activating airway DCs with an upregulation of inflammation, and heightened sensitivity in asthma. As a result of the loss of barrier integrity, infiltration of the epithelium by inflammatory cells, in the continuing presence of the injurious stimulus, alters cell structure and function leading to fibrotic modification, particularly in COPD (56). In a 3-year longitudinal gene chip analysis of transbronchial samples from 104 individuals with chronic bronchitis and 16 healthy control individuals, Samaha et al. (65) correlated gene expression with bronchial obstruction. They concluded that disturbances in the actin cytoskeleton facilitate bronchial inflammation and the transition from bronchitis. The inflammation is then mitigated by primary repair processes. Destruction of the bronchial matrix by hyaluronidase activity subsequently leads to reinforced inflammation and finally, matrix accumulation and organ fibrosis result in loss of function. Loss of surfactant also clearly plays a significant early role in this pathology.

### 4.2 Damage to surfactant and collectins

Lung surfactants are crucial to maintain stability and prevent collapse of the airway wall and improve bronchial clearance and regulate airway liquid balance. In addition, SP-A and SP-D provide initial defense against infectious agents and allergens, binding to some fungal and grass pollen allergens, suppressing IL-8 release from eosinophils and IL-2 from peripheral blood monocytes (17). Both SPs inhibit binding of specific IgE to glycosylated allergens from *Aspergillus fumigatus* and thereby block allergen-induced histamine release from human basophils (48). Mice genetically deficient in SP-A or SP-D were intrinsically hyper-eosinophilic, with marked increases in lung tissue levels of IL-5 and IL-13. These changes were generally reversed by intranasal replacement with the respective collectins, though SP-A replacement failed to reverse IL-13 levels. SP-D deficient animals were more susceptible to pulmonary hypersensitivity induced by *A. fumigatus* allergens and this could be reversed by intranasal SP-D or rhSP-D (66, 67). Oddly, the reverse was true with SP-A deficient mice, which had developed resistance to allergen-induced hypersensitivity and exhibited exacerbation of IL-5 and IL-13 levels and of pulmonary eosinophilia on intranasal replacement of SP-A. This suggests that SP-A and SP-D play somewhat different roles during lung allergy, SP-D being predominantly suppressive.

During an acute asthma attack, increased quantities of surfactant and phospholipids were detectable in sputum, indicating loss of surfactant, though this enhancement of surfactant loss was reversed during the recovery phase, presumably due to increased surfactant production, and loss of surfactant was not observed in stable asthma patients (68). Similarly, allergen challenge of patients with mild asthma led after 24 h to an increase in the bronchoalveolar lavage (BAL) levels of the structural collectins, SP-B and SP-C, but not in BAL phospholipids (69). The authors suggested that this reflects a distinction between acute release of collectin proteins, on the one hand, and subsequent loss of various surfactant components, on the other.

Increased oxidative stress damages surfactant phospholipids and either the resulting phospholipid hydroperoxides or the products of the neutrophil oxidative burst can also modify the structure of SP-A, so that it is no longer able to inactivate bacteria (19, 70). These mechanisms, together with the effects of inhibitory proteins, contribute to the functionally deficient airway surfactant in asthma (19).

Lung airway surfactants are also deficient because of cigarette smoking in COPD. There is both a loss of surfactant phospholipid into BAL and impaired surface activity of BAL surfactant (71). Cigarette smoking is further associated with a decrease in the BAL levels of lung collectins SP-A and SP-D (72), a change which exacerbates the dysfunctional surfactant barrier. This loss of SP-A and SP-D may be related to complex interactions with reactive oxygen species (ROS) generated by cigarette smoke.

Simple exposure of mice to inhaled ROS-generating 10 ppm ozone for 3 weeks resulted in severe pulmonary inflammation, with decreased expression of the antioxidant-enhancing Nrf-2 gene and increases in BALF and serum concentrations of both SP-A and SP-D (73). The inflammation induced by ozone was exacerbated in SP-D-/- animals. Intratracheal (i.t.) treatment of ozone-exposed mice with native amniotic SP-D once weekly for 3 weeks, markedly inhibited lung inflammation, improved lung function and increased lung Nrf-2 expression, and was associated with increased levels of SP-D in BALF but not in the sera of the animals. The lungs of SP-D treated animals revealed inhibition of ROS generation by the treatment. In support of this, SP-D was shown *in vitro* to inhibit H_2_O_2_-induced ROS generation, apoptosis and cell death in the A549 macrophage cell line, while enhancing Nrf-2 expression.

All these studies were also repeated by the authors in mice exposed for 6 weeks to inhaled cigarette smoke, as well as with murine bone marrow-derived macrophages exposed to cigarette smoke extract, giving similar results to those obtained with ozone, with one important exception. In the mice exposed for 6 weeks to cigarette smoke, the pulmonary inflammation was less pronounced than with ozone and SP-D levels were only raised in serum but not in BALF. This discrepancy may possibly be explained by the fact that while SP-D was shown to inhibit effects of pure ozone-generated ROS, ROS also adversely modify the structure of surfactant proteins. Thus, while not causing human SP-A degradation, ozone enhanced self-association and mannose-binding of SP-A, as well as reducing its ability to aggregate lipids and regulate alveolar type II cell and alveolar macrophage functions *in vitro* (74, 75). Ozone given *in vivo* also altered the ability of SP-A to modify alveolar macrophage function (76).

It would, thus, appear that in cigarette exposed animals and humans, the ability of SP-A and SP-D to modulate inflammation and promote surfactant and epithelial protection in the lungs is compromised by the alteration of their structure by ROS. Together with the degradation of SP-D by metalloproteinase (MMP) 9, discussed above (77), these modifications could also account for the difference in levels of SP-A and SP-D detected in the lungs and serum of patients with COPD or asthma, as discussed later.

In mice, specific deletion of the SP-D gene resulted in progressive chronic inflammation, emphysema and fibrosis. Already after 3 weeks, the lung tissue contained hypertrophic alveolar macrophages with increased expression of MMP2 and MMP9 and monocytic infiltrates. Hydrogen peroxide generation by alveolar macrophages was also markedly increased. Loss of SP-D production clearly elicits a vicious cycle of ultimately chronic lung injury (78).

Although SP-D and SP-A have critical roles in protecting the airway epithelium, they have also been used as biomarkers of epithelial barrier damage. In acute respiratory distress syndrome (ARDS), increased permeability positively correlates with serum SP-D, often associated with pulmonary oedema (79). Using LPS challenge in smokers and non-smokers as a model of ARDS, one of the smoking cohorts had exaggerated changes in barrier damage shown by increased serum SP-D and decreased BAL SP-D when compared to the non-smoking cohort (80). A decrease in BAL SP-D can subsequently impair proper protection and homeostasis of the epithelial barrier.

### 4.3 Alarmin and cytokine modification

Increased oxidative stress, with enhanced generation of ROS and a decrease in antioxidant mechanisms, also leads to increased alarmin and inflammatory cytokine generation (56). Under the influence of ROS generated by activated leukocytes present in COPD, IL-33 is oxidized, forming two disulphide bridges which alter the IL-33 conformation, so that it can no longer signal through ST2. Instead, IL-33ox binds to receptor for advanced glycation end products (RAGE) and EGFR to stimulate mucin production and decrease epithelial wound repair (81, 82). This indicates that IL-33-mediated immune defense is compromised by the inflammatory process in COPD.

IL-33, acting on ST2 receptors on Th2 lymphocytes, directly stimulates their chemotaxis and differentiation, thus contributing directly to asthma pathogenesis (83). In addition, though, IL-33 induces in bone marrow cells, the expression of granulocyte-macrophage colony stimulating factor (GM-CSF) which facilitates DC as well as granulocyte and macrophage expansion, which are integral to antiviral innate immune defense (84). In fact, treatment of mice with intranasal GM-CSF or genetic overexpression of the cytokine confers resistance to infection with influenza virus (85). GM-CSF is increased in sputum from COPD and asthma patients and its expression in bronchial submucosal tissue in asthma patients is enhanced (86). The increase in GM-CSF is especially pronounced after asthma exacerbations (87). This may promote the allergic asthma response since in mice, sensitized with HDM allergen, transient GM-CSF expression enhanced IL-33 levels generated by alveolar type II cells. Thus, there appears to be a complex mutual interaction between IL-33 and GM-CSF during allergic airway injury, promoting the allergic immune response, but also enhancing antiviral defense, despite the ongoing Th2-mediated allergic response.

In the lungs of GM-CSF deficient mice, alveolar macrophage differentiation is disturbed and there is an accumulation of surfactant proteins and phospholipids. This appears to be due to deficient catabolism of SP-A and phospholipids by the alveolar macrophages (88). As discussed later in relation to biomarkers (section 4.6), the interactions between GM-CSF and lung collectins are intricate. Thus, in SP-D deficient mice, local, LPS administration (i.t.) led to more pronounced lung inflammation than in wild-type animals, but with markedly reduced BAL GM-CSF concentrations, presumably due to removal of the LPS antagonistic and macrophage modulating effects of pulmonary SP-D. In contrast, in SP-D deficient animals treated systemically (i.p.) with LPS, despite clear lung inflammation, BAL GM-CSF concentrations were increased and survival of animals enhanced (89). Thus, while GM-CSF appears to regulate catabolism of local pulmonary surfactant and SP-A, the regulatory action of systemic SP-D on pulmonary inflammation appears to be dependent in some way—at least partially—on the activity of GM-CSF—at least in mice. Interactions between SP-D and GM-CSF are discussed further in section 4.6.2. in relation to cigarette smoke and COPD.

### 4.4 Epithelial signaling

#### 4.4.1 Role of interferons

While type I and III IFN generation is a cornerstone of immune defense against respiratory viral infection by activating an antiviral state in neighbouring cells, prolonged infection, with overexposure to the IFNs, causes increased inflammation, epithelial damage and inhibits the repair process (90). Consequently, epithelial cells become less responsive to IFNs, resulting with time in increased sensitivity to further infection (53, 91). This is frequently caused by chronic oxidative stress, often associated with chronic cigarette smoke in COPD. In asthma, bronchial epithelial expression of IFNs is additionally reduced, compromising immune defense even more (92).

#### 4.4.2 Pattern recognition receptors

Activation and modulation of the TLR and NOD-like receptor (NLR) pattern recognition receptor signalling pathways also occur in both COPD—related to cigarette smoking—and in asthma with triggering of cytokine release, enhanced inflammation and subsequent compromised adaptive immunity (62, 93, 94). Moreover, concomitantly with loss of response to IFNs during the ongoing airway inflammation in asthma and COPD, there is also loss of important protective mechanisms in epithelial cells.

Crucially, in habitual smokers and smokers with COPD, there is a downregulation of airway epithelial TLR expression, particularly of TLR5, which binds bacterial flagellin, as well as disturbance of TLR2 and TLR4 signaling, all contributing to the increased sensitivity to infections in COPD (95, 96). Also in COPD epithelium, there is loss of the polymeric immunoglobulin receptor (pIgR)/secretory component system, which ensures the transcytosis and release of polymeric immunoglobulins into mucosal secretions, with impaired antiprotease defenses and post-translational epigenetic enzymes, such as histone deacetylase 2 (HDAC2), and accelerated cellular senescence (97, 98). Many of these changes are probably related to enhanced ROS generation.

In contrast to COPD, expression of airway epithelial TLRs in asthma is enhanced, contributing to the pathogenesis of the disorder (99). For TLR4, which binds bacterial LPS, the rs4986791 genotype was shown to be significantly associated with severe asthma risk (100). A characteristic component of the airway dysfunction in asthma is the hypersecretion of the mucin MUC5AC, which leads to dyspnoea and coughing, representing a crucial cause of mortality in the disease (101, 102). Intriguingly, SP-D has recently been found to act directly on human bronchial airway cells and mouse airways *in vivo*, to inhibit MUC5AC secretion. The mechanism involves binding of SP-D to SHP-1 which ligates to SIRP*α* resulting in dephosphorylation of extracellular-regulated kinase (ERK) to inhibit MUC5AC secretion (103).

#### 4.4.3 Surfactant proteins

##### 4.4.3.1 Goblet cell hyperplasia and mucus secretion

Chronic inflammation and repeated injury lead to airway remodeling, resulting in altered epithelial differentiation. Goblet cell hyperplasia and mucus overproduction are hallmark features of allergic disease, and also often associated with COPD (56, 62, 104). In asthma, elevated Th2 cytokines, particularly IL-13 and IL-5 drive goblet cell hyperplasia via stimulation of ILC2 by alarmins, notably IL-25 and IL-33 (105). This is accompanied by altered mucin gene expression, particularly upregulation of MUC5AC. In an acute mouse allergic asthma model using ovalbumin, administration of IL-25 blocking antibody significantly reduced IL-13 and IL-5 levels, resulting in decreased mucus production, eosinophil infiltration, and goblet cell hyperplasia (106).

In COPD, similar features of goblet cell hyperplasia are present, and mucus hypersecretion occurs, though the mechanisms differ. Epithelial injury caused by cigarette smoke or pollutants promotes IL-33 release, causing increased IL-13 production, while decreased mucociliary clearance exacerbates mucus retention (104). Models with deficient SP-D present with pulmonary emphysema, and exacerbated inflammation including accumulation of foamy macrophages (73, 107). Taken together, it is apparent how damaging epithelial dysfunction, exemplified by an impaired barrier, disrupted innate immunity, and excess mucus, contributes to the chronic inflammation of asthma and COPD.

### 4.5 Modified immune response

While there is wide agreement that the epithelial immune response to viral infection and sensitivity to IFNs during chronic airways inflammation is disturbed, the extent to which the generation of defensive IFNs are affected, at least in asthma, is not entirely clear (108).

Using differentiated airway epithelial cells from healthy, COPD, and asthma subjects, Veerati and colleagues (109) assessed rhinovirus-induced innate immune responses by determining gene expression of IFN (type-I, II, and III), IFN response factors (IRF1, IRF3, and IRF7), TLR signaling and NF-κB and signal inducer and activator of transcription-1 (STAT1) activation. They found that while gene expression in healthy epithelial cells peaked around 48 h after viral infection, in both asthma and COPD cells, maximal gene expression was observed 72–96 h post infection or even later. They concluded that the local inflammatory response in the lung altered the responsivity of epithelial cells (even in the absence of inflammatory cells) leading to a delay in the initiation of the IFN antiviral response. This may have been related to the predominant disease phenotype cytokines (e.g., type 1 or 2) of the patients from whom the cells had been obtained. The mechanisms involved in the delay to the IFN response, though, are likely to be multiple. This is illustrated by the recent finding in mice, that a prior allergic response induced by HDM allergen was associated, in lungs from mice subsequently exposed to influenza A virus, with less upregulation of Th1-related genes and antiviral pathways, suggesting both impaired Th1 immunity and suppressed local antiviral responses (110). Attempts to develop inhaled IFN as a therapeutic for acute exacerbations have so far been unsuccessful, likely, at least in part, due to the challenges of conducting clinical trials in this setting (111).

Similarly, several weeks of exposure of mice to cigarette smoke, resulted during subsequent infection with influenza A virus, in suppression of IL-6 and IFN gene expression in lung tissue, prolonged neutrophil infiltration and inflammation with dysregulation of macrophage activation, associated with slower virus clearance (112). This is likely to be of relevance for the increased susceptibility of COPD patients to infections.

The increasing use of single cell transcriptomics has revealed both an unexpected diversity of cell types in the airway epithelium and the complex interactions between these cells and innate and adaptive immune responses in the lung. Complexity and plasticity are further enhanced by pathogens and stimuli to airway disease (30). We provide some insights below.

#### 4.5.1 Complexity of response to allergens

For instance, secreted IgA (sIgA), generated in response to antigens by mucosal lymphoid tissue, is taken up by epithelial cells and secreted into the mucus. If the sIgA does not crosslink with antigen, it has a suppressive effect on eosinophils and basophils, but once cross-linked under antigen exposure, the cells are activated (113). In addition, epithelial cells with gene characteristics of both mucous and ciliary cells, called mucous ciliary cells, can be detected in asthmatic patients (25) and chemosensory, tuft-like cells have been identified as a major source of epithelial IL-25 in chronic rhinosinusitis with nasal polyps (114). Furthermore, at least in mice, colony stimulating factor-1 (CSF-1) generated by airway epithelial cells, in response to allergen, binds to CSFR-1-expressing alveolar DCs, enhancing allergen presentation and the promotion of type 2 immunity (115).

Thus, the airway epithelium is intimately involved in the local immune response during asthma (113). On the other hand, the local immune response generated by external stimuli can also feedback on the epithelial cells. In this respect, type I and III IFNs, generated as part of the host defense response by resident immune cells in the airway epithelium, can suppress epithelial repair if pathogen removal is delayed (30). Also, with regard to asthma, SP-A binds to various pollens and both SP-A and SP-D bind to oligosaccharides on dust mite allergens, thereby inhibiting the binding of allergen-specific IgE and reducing humoral immune responses (116).

#### 4.5.2 SP-D involvement

The disease-induced changes in epithelial function are closely linked with production of SP-D. Thus, in children with acute asthma, salivary SP-D levels were higher than in healthy children and were further raised during an acute asthma attack, being correlated with predicted severity of exacerbation, indicating increased involvement of SP-D during the exacerbation (117). However, in a comparative study of SP-D levels in BALF and serum from mild and severe asthmatic patients, MacKay et al. (118) found that BAL SP-D levels in severe asthma were significantly lower than in healthy controls or mild asthma. This decrease was associated with increased BAL neutrophil counts and LPS concentrations and inversely correlated with eosinophilic cationic protein concentrations. In contrast, circulating serum SP-D levels were significantly increased in severe vs. mild asthmatics and associated with degraded SP-D fragments. The authors suggested that the epithelial dysfunction in severe asthma, as reflected also by decreased BAL SP-D levels, indicates defective innate immunity within the airways. A recent meta-analysis of 16 studies conducted by Mohamed and colleagues investigating asthma severity and SP-D revealed a trend of slightly elevated serum and sputum SP-D levels in asthmatics when compared to non-asthmatics (119). The findings, though, were found to be non-significant. However, there do not appear to be any studies linking frequency of infectious exacerbations with decreased local SP-D levels in asthma.

Epithelial-derived SP-D, in addition to allergen masking, inhibition of allergen-IgE interactions and promotion of alveolar macrophage and DC phagocytosis, is also able to regulate apoptosis of activated eosinophils, as well as exerting direct effects on B and T cell proliferation, resulting in skewing of Th2 to Th1 responses (27). Thus, SP-D is a component of the regulation of macrophage efferocytosis of different apoptotic cells during lung inflammation. Recent studies indicate that during the resolution phase of an acute non-infective lung inflammation, clearance of a superimposed bacterial infection is suppressed in alveolar macrophages subjected to apoptotic neutrophils but enhanced by apoptotic epithelial cells (120). In an IL-4/IL-13-enriched environment, as in asthma, apoptotic neutrophils induced a tissue remodeling macrophage phenotype and apoptotic epithelial cells induced a tolerogenic macrophage phenotype (121). In view of the phagocytosis-promoting and macrophage-modulating effects of SP-D, it will be of interest to what extent this collectin affects the macrophage efferocytotic and phenotyping process during lung inflammation.

#### 4.5.3 Apoptosis

Epithelial basal cells are now known, like alveolar macrophages and DCs, to phagocytose apoptotic epithelial cells, probably via the AXL receptor tyrosine kinase which binds growth factors and is involved in cell proliferation and differentiation. In early COPD, these cells undergo hyperplasia and may modulate inflammation in response to the enhanced apoptosis of cells (30, 122). In chronic rhinosinusitis, these hyperplastic basal cells exhibit upregulated genes that are responsive to IL-4 and IL-13, indicating an enhanced sensitivity to type 2 immune responses (123). It is possible that SP-D may play a role in these functions of basal cells (27).

#### 4.5.4 IL-33 modification

In addition to loss of ST-2 responses, due to oxidation of IL-33 by ROS, the action of IL-33 on ST2 receptors is subject to modification by cigarette smoke. Studies in mice have shown that while initially stimulating IL-33 release, cigarette smoke subsequently decreases ST2 expression on ILC2s and increases ST2 expression on natural killer (NK) cells and macrophages in mice subject to influenza virus (124). As a result, Th2 responses are dampened and type 1 immune responses to viruses exaggerated, with increased IFN-γ release from epithelial cells (125). These changes promote further epithelial injury, compromising and delaying epithelial immune defense and facilitating pathogen invasion and chronic inflammation, thus contributing to disease progression. This modification of IL-33 action probably contributes in COPD patients to impaired innate immune responses.

### 4.6 Biomarkers of epithelial dysfunction

Epithelial-derived biomarkers can serve as indicators of disease severity and progression in COPD. Notable biomarkers include the immunomodulatory, non-ciliated, bronchial epithelial derived marker, club cell secretory protein (CC-16), reduced circulating levels of which indicate epithelial injury, and SP-D. Both CC-16 (previously termed CC-10) and circulating SP-D have been implicated in a variety of studies as biomarkers of lung injury and clinical outcome in COPD (126). CC-16, though, is negatively associated with the severity of asthma (127).

#### 4.6.1 Collectins

It will be clear from what has been described so far that BAL collectins are raised after injurious stimuli and subsequently decrease, while serum SP-D increases (128). Importantly, as also discussed above, SP-A and SP-D are both generated from pulmonary and extrapulmonary sites (128, 129). However, as a result of lung injury or tobacco smoke, both SP-A and SP-D are translocated from BAL into the serum, because of increased alveolar-capillary leakage and the partial decomposition of multimeric collectins to smaller molecular forms (21, 128). Consequently, much of the circulating SP-A and SP-D in smokers and COPD patients consists of various molecular forms derived from the inflamed lungs.

#### 4.6.2 COPD

In a long-term longitudinal study on 538 twins, it was found that serum SP-D was significantly higher in smokers than in non-smokers. Among the smokers with a high baseline serum SP-D concentration, there was a significant decrease over the 12 years in lung function (FEV_1_) in comparison to non-smokers (130). Among 36 individuals with normal FEV_1_ at baseline, who subsequently developed COPD, a higher baseline serum SP-D concentration tended to be associated with a greater decline in FEV_1_.

The distinction between the effects of SP-D generated locally in the lung and systemically active SP-D was investigated in normal mice and SP-D deficient (Sftpd-/-) animals treated either with intratracheal (i.t) or intraperitoneal (i.p) bacterial LPS, at doses ensuring similar LPS levels in BALF in each case (89). Assessing lung inflammation after 6 h or 16 h in terms of IL-6 concentrations in BALF, inflammation was greater in Sftpd-/- than in normal mice, but the increase in BALF IL-6 in indirectly (i.p) injured animals, surprisingly, was 10-fold greater than in directly (i.t) injured mice. While infiltrating neutrophils dominated in lungs of directly injured Sftpd-/- mice, monocytes/macrophages were predominant in indirectly injured Sftpd-/- mice. This difference was associated with a significant increase in GM-CSF in BALF after i.p. LPS, but a marked, significant fall in GM-CSF after i.t. LPS. The authors concluded that (in the mouse, at least) under an indirect, systemic inflammatory stimulus to the lungs, SP-D inhibits monocyte/macrophage infiltration into the lungs by reducing GM-CSF generation, while acutely released local SP-D had a minor effect on the lung inflammation. If this is also true for humans, systemically generated SP-D may be of importance in modulating inflammatory lung disease and by extension, SP-D may be of therapeutic benefit.

Levels of SP-A and SP-D in BAL fluid obtained from smokers are both significantly reduced in comparison to levels in BAL fluid from non-smokers (72). This indicates that local airway epithelial production of both immune enhancing collectins is compromised during cigarette smoking. Since SP-D is important for the regulation of the metabolism of epithelial phospholipids, important components of lung surfactant, loss of SP-D during smoking may also result in loss of protective membrane surfactant (131, 132).

In an extensive study of SP-D levels in BAL fluid and serum in smokers with COPD, lower levels of SP-D were found in the lungs, in comparison to both otherwise healthy smokers and non-smokers, while serum SP-D was higher than controls in COPD smokers. Serum SP-D levels correlated with the degree of airway obstruction in all smokers (133). Interestingly, serum SP-D consisted mainly of small molecular sub-units. The authors proposed that due to local degradation in the lungs, the smaller subunits were able to translocate across the more permeable epithelium and enter the circulation, accounting for the higher serum levels. Any use of serum SP-D as a serum biomarker in COPD should, therefore, take into consideration the chemical structure of the molecule.

Exhaled SP-A particles, reflecting locally produced collectin in the lungs, were also reduced in a pilot study in patients with stable COPD in comparison to healthy subjects (134). Interestingly, while the densities of both alveolar macrophages and type 2 pneumocytes in lung tissue from 32 male COPD patients increased, the densities of SP-A positive type 2 pneumocytes decreased, while SP-A positive macrophages increased in correlation with airway obstruction (FEV_1_) (135). This suggests that with SP-A at least, the decrease in pulmonary levels reflects injury to type 2 epithelial cells.

As pointed out by Watson et al. (23), partially due to leakage from the lung, elevated plasma levels of SP-D have been shown to correlate with the early phase of COPD exacerbations in AECOPD and indices of lung function, including FEV_1_ and the BODE (body mass index, airflow obstruction, dyspnea, exercise capacity) index in stable COPD (136, 137). Consequently, increased plasma SP-D in patients with chronic COPD may reflect acute exacerbation of the disease.

#### 4.6.3 Asthma

Allergen challenge of patients with mild asthma was reported, after 24 h, to have no effect on BAL SP-A, but to increase the BAL level of SP-D and SP-C, which both correlated with BAL eosinophil numbers (69). In other studies, in patients with mild asthma, though, BAL levels of both SP-A and SP-D were increased in comparison to control patients (21, 51). Multiple studies have observed increased serum levels of SP-A and SP-D in asthmatic patients, but at times, contradictory findings, particularly with SP-A (21). This may reflect degradation of the collectins during pulmonary inflammation and conversion to low molecular forms, as discussed below.

#### 4.6.4 SP-D enzymatic degradation

The induction of airway inflammation, while promoting immune defense reactions, if sustained, subsequently leads to modulation of epithelial defense. A commonly generated mediator during airway inflammation is the enzyme MMP9, a tissue protein degrading, chemotactic enzyme generated by a variety of leucocytes, as well as lung epithelial and endothelial cells, which has been shown to cleave SP-D *in vitro* (77). As a result, bacterial aggregation and opsonization and enhancement of macrophage phagocytosis by SP-D are suppressed. Since MMP9 concentrations are raised in sputum and serum of smokers and in patients with airway inflammation, including those with COPD or asthma, the enzyme is likely to affect the airway levels of SP-D (77). Neutrophil elastase (NE), released by activated neutrophils, is also a key enzyme capable of cleaving SP-D and SP-A. In airway diseases with high neutrophil infiltration, such as COPD, NE can reduce the agglutination capability of SP-D (138, 139). Similarly, cathepsins, and proteases generated by bacteria are also reported to cleave SP-D and SP-A. These degradation processes emphasize that implementation of collectin assays as biomarkers requires distinction between molecular forms (138, 140).

#### 4.6.5 SP-D polymorphisms

Because of the polymorphism in the rs721917 SNP genotype of the SP-D gene, a more recent study has assessed the levels of high and low molecular weight SP-D in serum of Lebanese patients with COPD or asthma (141). While serum SP-D levels were raised in COPD patients, this was not the case in asthma patients. Irrespective of whether healthy or suffering from respiratory disease, Met11/Met11-genotyped individuals had the highest serum SP-D levels and Thr11/Thr11-genotyped individuals the lowest. In healthy individuals, the former consisted of the multimeric and the latter of the smaller subunit forms. While the rs721917 polymorphism was not predictive for either COPD or asthma, both respiratory conditions were associated with a significant, relative reduction in serum low molecular, multimeric SP-D levels in Met11/Met11individuals. The authors concluded that degradation processes arising from the lung inflammation were crucial in determining the levels of serum SP-D detected, but that future studies on serum SP-D as a biomarker would benefit from distinction between multimeric and smaller molecular forms of the collectin. It would seem likely that determination of trimeric and monomeric forms of serum SP-D would reflect more accurately the degree of pulmonary epithelial injury.

Consequently, SP-D—particularly monomeric and degraded forms, and to some extent SP-A—would appear to be complementary to the circulating alarmins IL-33 and TSLP as biomarkers of lung epithelial injury and inflammation (142, 143). IL-33, through interaction with its receptor ST12, has been associated with Th2-mediated immune responses in patients with asthma and increased levels of this alarmin have been detected in lung epithelial cells and serum of patients with asthma. Moreover, in keeping with an etiological role in this disease, single nucleotide polymorphisms in the IL-33 gene predispose to the development of asthma (143). Similarly, in COPD, both increased expression of IL-33 and the ST2 receptor has been observed. Cigarette smoke, a key inducer of COPD, not only activates IL-33 production by epithelial and endothelial cells but also induces the expression of IL-33 in peripheral blood mononuclear cells (143). Identification of these biomarkers, thus, allows for better disease monitoring and therapeutic targeting in a variety of chronic respiratory inflammatory disorders.

## 5 Implications for therapy of respiratory inflammation

### 5.1 Targeting epithelial barrier integrity

Enhancing epithelial repair and barrier function is a key therapeutic strategy for chronic respiratory diseases. Several approaches are currently being taken.

#### 5.1.1 Short-chain fatty acids

In addition to the crucial importance of surfactant phospholipids in maintaining lung epithelial barrier function, increasing evidence indicates that other endogenous lipids also appear to be important. Short-chain fatty acids, SCFAs (propionate and butyrate) have been shown to maintain airway epithelial barrier function. These two lipids are generated in the gut microbiome by the action of resident bacteria and are transported in the blood to the lung (144).

In the gut, SCFAs play a variety of roles in energy metabolism and modify cell signaling pathways by binding to cell surface receptors including G-protein-coupled receptor 41 (GPR41), GPR43, GPR109A, peroxisome proliferator-activated receptors *γ*, and aryl hydrocarbon receptor (AHR). In both gut and lung, the SCFAs promote epithelial barrier function and facilitate resolution of inflammation (144). For instance, induced deficiency of AHR and thus, also reduced production of SCFAs in mice, led to increased lung epithelial leakage, leukocyte infiltration and lung injury in response to influenza A virus infection. Genetic promotion of AHR activity or dietary supplementation with the AHR ligand indole-3-carbinol, on the other hand, both reduced virally induced epithelial dysfunction (145). The SCFA butyrate directly affects the differentiation of epithelial cells, phagocytes, B cells and plasma cells, and regulatory and effector T cells and thus modifies both epithelial function and inflammatory responses (146). In phagocytes, it also suppresses the oxidative burst and the Notch-mediated generation of ROS by epithelial cells, leading *in vivo* to reduced epithelial injury (147, 148).

In agreement with these findings, dietary treatment of rats with butyrate prevented hypoxia-induced right ventricular hypertrophy (RVH), hypoxia-induced increases in right ventricular systolic pressure (RVSP), pulmonary vascular remodeling, and permeability (149). In mice with cecal ligation and puncture (CLP) induced sepsis, both intestinal and lung epithelium is damaged. Treatment of CLP septic mice with butyrate inhibited lung inflammation and improved epithelial intercellular tight junction expression, reduced BALF levels of proinflammatory cytokines, and—of relevance to the current article—raised levels of SP-D, while increasing the levels of anti-inflammatory cytokines (150). Furthermore, the CD4+/CD8+ T-cell ratio and the proportion of CD4 + Foxp3 + regulatory T cells (Tregs) were increased by sodium butyrate. These data indicate that butyrate mitigated inflammatory lung injury in CLP mice but also promoted immune defense responses, including enhancement of lung SP-D levels. Beneficial actions of dietary butyrate have also been observed in mice with ovalbumin-induced allergy. These were predominantly marked by stimulation of FoxP3 in T regulatory cells, with resulting suppression of Th2 and Th9 cell responses and the associated pulmonary eosinophil infiltration (151).

A recent meta-analysis of 37 randomized controlled trials involving 1975 COPD patients and 21 different nutritional supplements, reported that butyrate, nanocurcumin and probiotics significantly improved FEV_1_ (152). Nanocurcumin ranked highest for improving FEV_1_/FVC (Forced Vital Capacity). Clearly, dietary butyrate and some other nutritional supplements may provide supportive treatment in decreasing airway epithelial permeability and maintaining barrier function during pulmonary inflammation.

#### 5.1.2 Myosin light chain kinase 1 (MLCK1) inhibition

MLCK1 plays a crucial role in regulating lung and gut epithelial barrier function by controlling tight junctions. It is a protein kinase that phosphorylates myosin light chain (MLC), which then interacts with actin filaments, causing contraction of the perijunctional actomyosin ring, a structure critical for maintaining epithelial barrier integrity (153).

Infusion (i.t.) of LPS from *Pseudomonas aeruginosa* enhanced airway epithelial paracellular permeability in parallel with leukocyte infiltration and an increase in phosphorylated myosin light chain (p-MLC) levels in bronchial tissue explants. These changes were associated with an increase in the percentage of tight junction opening between ciliated cells 4 h after *in vivo* LPS administration. In NCI-H292 cells, p-MLC levels were also increased after exposure to LPS from *E. coli* and this increase was inhibited by the MLC inhibitor 5-iodonaphthalene-1-sulphonyl-homopiperazine (ML-7) (154). Pretreatment of rats for 2 days with ML-7, followed by *P. aeruginosa* LPS i.t. inhibited p-MLC levels and tight junction opening in bronchial tissue, as well as blocking the changes in airway epithelial permeability and leukocyte infiltration. In a later study, intratracheal administration of ML-7 or ablation of MLC significantly promoted neutrophil apoptosis, accelerating their clearance. In the LPS- or CLP-induced sepsis models, ML-7 administration significantly increased apoptosis of neutrophils at the infection site but not in circulating neutrophils. ML-7 also significantly improved the survival rate of mice with both types of sepsis (155). The authors suggested that ML-7, in addition to its actions on epithelial cells, increases leukocyte apoptosis and the lysosomal clearance of the cells. Other MLCK1 inhibitors, including myokinasib-II and the MLCK1 membrane recruitment inhibitor divertin have been reported, but while divertin has also been shown to inhibit gut epithelial permeability and disease in animal models of inflammatory bowel disease, no effects on lung inflammation or clinical development activities have been reported (156, 157). Nevertheless, MDCK-1 inhibitors remain a therapeutic possibility for epithelial barrier dysfunction.

### 5.2 Modulating epithelial-based innate immune responses

In addition to the supportive benefit of nutritional supplements, a variety of potential therapeutic approaches are being taken to promote endogenous immune defense responses to combat infectious pathogens during infections and in chronic airways inflammation (34). Preservation of defense mechanisms while reducing excessive immune activation and tissue injury is crucial for effective therapy of airway inflammation. Strategies include inhibitors of the epithelial alarmins, PRR modulators to targets like TLRs, and anti-inflammatory biologics, mainly targeted towards inflammatory cytokines and alarmins.

#### 5.2.1 Pattern recognition receptors (PRRs)

Epithelial cells express PRRs that recognize pathogens and DAMPs, triggering immune responses. These include the TLRs, TLR2-9, which have been shown to play roles in both recognition of pathogens and in the modulation of inflammation during asthma and COPD. Two TLRs that have received interest in this context are TLR2 and TLR4 (158, 159) with INI-2004 a TLR4 agonist currently undergoing clinical development in allergic rhinitis (NCT06038279). TLR7 agonists AZD8848 (160) and GSK2245035 (161) have entered clinical development for airways disease but have been discontinued. A recent TLR targeting agonist is INNA-051, a TLR2/6 targeting respiratory viral infections (162). Given prophylactically and intranasally, INNA-051 has shown promise against influenza, RV, and SARS-CoV-2 by preventing viral entry into epithelial cells via early stimulation of the innate immune response (163).

#### 5.2.2 Cytokine/alarmin modulators

Several antibody therapies targeting soluble mediators are currently licensed for the treatment of airway inflammatory diseases, including anti-IL-5 antibodies mepolizumab, benralizumab, and reslizumab, the anti-IL-4/-IL-13R dupilumab, and the anti-TSLP antibody tezepelumab. The latter has also been shown to be of benefit in the epithelial-driven disease, chronic rhinosinusitis with nasal polyps (142).

In addition, IL-33 inhibitors or antagonists are under clinical development (125, 164). Sanofi's IL-33 antagonist, itepekimab, has been shown in one phase III clinical trial (AERIFY-1) to reduce exacerbations in COPD by 27%, but a second study (AERIFY-2) failed to show benefit which was partly explained by the company due to the lower than expected exacerbation rate during the studies (165). Astegolimab (Roche/Genetech) anti-ST2 failed to meet the primary endpoint (COPD exacerbation reduction in a recent phase III study (ARNASA) although in a phase 2b study (ALIENTO) a small (15.4%), but statistically significant, reduction in COPD exacerbations was seen (166). AstraZeneca's IL-33 blocker, tozorakimab is in phase III clinical trials with primary endpoint reduction in COPD exacerbations. Tezepelumab, while licensed for asthma, is also undergoing phase III trials for COPD exacerbations (164). The strategy behind most of these studies is to reduce inflammation and exacerbations.

#### 5.2.3 Inhaled corticosteroids (ICS)

ICS such as fluticasone proprionate, fluticasone furoate, beclamethasone diproprionate, budesonide, with or without concomitant β-adrenoceptor agonists to relax bronchial smooth muscle, are a standard treatment for asthma, reducing inflammation by activating glucocorticoid receptors and suppressing the production of inflammatory mediators—including cytokines and alarmins. In COPD, ICS are used to prevent exacerbations, but they are not effective in all patients. Moreover, corticosteroids have very broad anti-inflammatory actions, also inhibiting adaptive immune responses and exerting adverse actions. In fact, fluticasone propionate has been shown to suppress anti-viral immunity and to increase bacterial loads and mucus production in COPD patients (167). Consequently, in both asthma and COPD, ICS enhance the risk of respiratory infections and pneumonia. Since cigarette smoking leads to the reduction of viral clearance observed in COPD, the use of ICS in COPD is further limited (112).

ICS have been associated with increased SP-D levels, suggesting a potential mechanism through which these medications exert their anti-inflammatory effects. In a study in 20 subjects with COPD, former smokers with COPD had significantly lower SP-D levels in BAL than healthy subjects (168). COPD was independently associated with lower SP-D levels and inhaled corticosteroid use was independently associated with higher SP-D levels. Treatment of type II alveolar epithelial cells isolated from adult rat lungs with dexamethasone resulted in a significant increase in SP-D mRNA and protein production after 4 days of culture.

#### 5.2.4 Collectins

In keeping with the studies discussed above on the suppressive effects of SP-D on responses to *A. fumigatus* allergens, intranasal treatment of mice with invasive pulmonary aspergillosis with SP-D or rhSP-D markedly reduced mortality over a period of 15 days (169), suggesting its possible therapeutic use in the treatment of allergic responses or asthma. Moreover, the recent discovery of the inhibitory action of SP-D on MUC5AC secretion may offer a novel approach to the mucin hypersecretion characteristic of airway diseases (103).

A full-length recombinant form of human SP-D, zelpultide alfa (AT-100), is currently in phase III clinical testing by Airway Therapeutics as an orphan drug for prevention of bronchopulmonary dysplasia (BPD) and minimization of resulting lung damage in preterm infants (170, 171).

The PDE4 inhibitor roflumilast, when added to cultures of alveolar epithelial type II cells, caused a minor increase in SP-A, SP-C and SP-D gene expression, particularly when cyclic AMP levels were pharmacologically enhanced by adding prostaglandin E2 in the presence of indomethacin (172). The authors suggested that SP-D generation may contribute to the therapeutic benefit of roflumilast.

#### 5.2.5 Macrolides

The macrolide antibiotic azithromycin exhibits a range of immunomodulatory and anti-inflammatory properties, including protective actions on bronchial epithelial cells, and is included in the Global Initiative for Chronic Obstructive Lung Disease (GOLD) guidelines as an option to reduce exacerbations of COPD (173). While its antibiotic activity may contribute to its therapeutic benefit, its efficacy in chronic respiratory diseases is probably due more to its anti-inflammatory/immunomodulatory actions. Administered for 12 months, azithromycin has also been shown in a randomized, double-blind, placebo-controlled trial (174) to reduce exacerbations and improve quality of life with azithromycin included as a therapeutic option in treatment guidelines (175).

Aside from COPD, azithromycin has proven effective in asthma patients. Long-term, low dose azithromycin was shown to reduce exacerbation frequency and improve quality of life in patients with severe asthma regardless of type (174), and patient remission was achieved in both eosinophilic and non-eosinophilic asthma (176). Using samples from a sub-population of the previously mentioned AMAZES trial, azithromycin treatment suppressed dysregulated TNF signaling components (177). It is apparent that multiple epithelial-immune mechanisms are involved in the beneficial effects of macrolides in both asthma and COPD, which are explored in several reviews (173, 178).

Azithromycin reduces, probably by actions on host cell responses, the *in vitro* replication of a number of viruses, including rhinoviruses, influenza A and coronaviruses, and has been reported to exert antiviral effects in a limited number of clinical studies (179, 180). In this context, several studies have indicated that azithromycin enhances viral-induced IFN-β generation by human airway epithelial cells *in vitro* (181). Consequently, quite apart from its antibacterial activity and the resulting adverse effect of promotion of antibiotic resistance, azithromycin has the benefit of early activation of epithelial host defense against infection with subsequent inhibition of damaging inflammation. As a result, there is increasing interest in the relevance of azithromycin treatment for facilitation of epithelial immune defense.

The non-antibiotic azithromycin derivative glasmacinal (EP395), under development for the prevention of COPD exacerbations, has similar immunomodulatory and anti-inflammatory actions to those of azithromycin and has also been shown recently to have a comparable effect on epithelial IFN-β generation *in vitro*, in association with a decrease in rhinovirus load (182, 183). In healthy participants, glasmacinal pretreatment acutely enhanced serum SP-D concentrations, while decreasing inflammatory cytokines in BALF with inhaled LPS challenge, suggesting that it was promoting epithelial immune defense (184). Glasmacinal, thus, seems to retain the initial promotion of epithelial immune defense responses, as well as the later anti-inflammatory effects of azithromycin, but without antibacterial activity and the associated risk of bacterial resistance.

### 5.3 Precision medicine and biomarker-driven therapies

Advancements in biomarker profiling allow for personalized therapy in COPD and other airway diseases. Stratification of patients based on biomarker expression, development of targeted biologics for specific inflammatory endotypes, and use of exosomal biomarkers for early detection and prognosis are potential strategies.

Blood eosinophil count is being increasingly used, not only in asthma as a type II eosinophil driven allergic disease, but also in COPD. In the latter condition, eosinophilia is a valuable biomarker to predict both prognosis and treatment response and is used to adjust the use of corticosteroids for maintenance therapy and during an exacerbation (185) as well as appropriate patient selection for biological therapies, for example, dupilumab and mepolizumab. In the light of the discussion in previous sections of this review, the use of serum SP-D and its degraded forms is a potential biomarker. As discussed in section 4.6, SP-D is being increasingly considered as a biomarker for chronic bronchial epithelial injury. However, its practical use in the acute setting, such as therapeutic response in acute exacerbation of airways disease or monitoring response of therapies that enhance immune defense to acute infection, is likely to be limited due to the fact that timing of the sample is critical and therefore, practically challenging to use in the real world setting.

### 5.4 Regenerative cell-based therapy

Since licensed therapies for COPD are mainly those alleviating bronchial smooth muscle or inflammatory responses, rather than the underlying tissue damaging pathologies, attempts are being made to assess the value of mesenchymal stem cells to promote tissue repair. A recent review and meta-analysis has addressed the outcome of 11 studies in 330 patients with COPD treated with MSC mesenchymal stem cells (186). The assessment concluded that while regenerative cell therapies significantly improved exercise capacity in COPD patients (6 min walking test) and produced a trend for improvement in FEV_1_, hospitalizations due to exacerbations were not reduced. However, the studies considered were all small with insufficient numbers of patients and different designs, thus limiting the weight of the evidence. Clearly, more intensive clinical studies are needed on the value of regenerative therapies in the treatment of chronic lung inflammation.

## 6 Conclusion

The airway epithelium plays a fundamental role in lung homeostasis, acting as both a barrier and an immune sentinel. In COPD, epithelial dysfunction contributes to chronic inflammation and disease progression. This, in turn, leads to deficient epithelial immune defense responses to pathogens. Understanding epithelial-immune interactions and the roles of collectins opens new avenues for therapeutic intervention, emphasizing the parallel importance of barrier restoration, maintenance of immune defense responses, immune modulation, and biomarker-driven precision medicine. Future research should focus on translating these insights into effective clinical strategies for managing airway diseases.
